# Knowledge Needs in Rehabilitation: Perspectives of Service Users and Professionals

**DOI:** 10.3389/fresc.2022.858081

**Published:** 2022-05-13

**Authors:** Salla Sipari, Mia Tammelin, Sari Helenius, Eija Janhunen, Merja Rantakokko, Nea Vänskä, Toini Harra

**Affiliations:** ^1^Customer-Oriented Wellbeing and Health Services, Metropolia University of Applied Sciences, Helsinki, Finland; ^2^School of Health and Social Studies, Institute of Rehabilitation, JAMK University of Applied Sciences, Jyväskylä, Finland; ^3^School of Rehabilitation and Examination, Metropolia University of Applied Sciences, Helsinki, Finland

**Keywords:** knowledge, implementation, rehabilitation, applied research, practices

## Abstract

Applying rehabilitation research knowledge in practice is challenging due to a gap between scientific knowledge produced by researchers and the needs of practical rehabilitation. This study describes the current and future knowledge needs of rehabilitation research from the perspectives of professionals and service users. We conducted a qualitative study with inductive content analysis from nine focus group interviews with rehabilitation stakeholders. The results show that current knowledge needs are strongly related to the meaningful and inclusive life of service users, the promotion of multi- and interprofessionalism in rehabilitation, and transdisciplinary applied research on rehabilitation. The future knowledge needs were related to the changing needs of rehabilitation and remote rehabilitation based on rapid change in society and digitalisation and on different rehabilitation practices and contexts. The results of the study can be used to enable favorable conditions for reciprocal research, development, and innovation (RDI) activities and research networks in transdisciplinary rehabilitation.

## Introduction

In Finland, good rehabilitation practice is based on the needs and the individually tailored rehabilitation plan of a service user (SU) as well as on the knowledge from empirical practice and evidence of effective rehabilitation from research (1, 2). However, the implementation of research results is demanding, and translations of research findings into rehabilitation practice are slow (3, 4). A particularly critical factor in implementation is understanding the complexity and process-nature of applying the relevant study results in the context of practical rehabilitation (3). Concerns have been raised that research does not reflect the realities or needs of SUs (5, 6).

In order to apply the evidence from research to rehabilitation, the importance of many underlying factors must be understood. Rehabilitation is an interactive process in which the relationships between professionals and SUs are crucial and collaborative. According to Vingerhoets, Hay-Smith, and Graham (7), it is crucial to look at the intersection of SUs' values, professionals' expertise, and evidence from research. People who take part in the collaboration enrich the work by bringing their own unique perspective and different experiences to it. Therefore, attention needs to be paid to the knowledge and skill needs and attitudes of participants in order to create evidence-based practice (EBP), which means integration of the scientific evidence, clinical expertise and individual and family preferences (8). This can be enhanced by collaborating and rejoining different perspectives together at the intersection of values, expertise, and evidence.

According to Sarkies et al. (4), EBP implementation and the translation of knowledge into practice are widely studied areas. One recognized barrier for EBP is that the implementation phase only starts at the end of the research process and is the responsibility of the researchers. One aspect of promoting the implementation and uptake of information from research is to look at it from the perspective of users of that information. It appears that little research has been done on the information needs of rehabilitation professionals and SUs.

Integrated knowledge translation (IKT) is a research method that brings together researchers and stakeholders with experience and knowledge of a research topic. Within IKT, partnerships have been developed that enhance the relevance of research results among rehabilitation professionals and SUs (5). In this kind of development, the focus is both on the possibilities of knowledge utilization and the knowledge needs in daily rehabilitation practice from the perspective of professionals and SUs. In order to not only translate the research results into rehabilitation practice, but also to create usable knowledge in collaboration based on evidence from research, it is crucial to understand the knowledge needs of professionals and SUs in practice.

In Finland, rehabilitation is financed through several channels, mainly in accordance with the Nordic welfare state principle with public funds, but also through insurance companies and private financing. The rehabilitation system is decentralized to many areas, such as health care, social care, school, labor administration, and prison management (9). Because rehabilitation as a phenomenon is divided into several fields, rehabilitation research has also been fragmented across many fields. Approaches in different research fields vary, and thus the language of research also varies and depends on the field in which the research is conducted ((10): 3). The fragmentation of the rehabilitation field and the diversity of the research concepts make cross-sectoral collaboration and the defining of common concepts difficult ((11): 31).

In the field of rehabilitation, multidisciplinary research have been discussed for over 30 years (12). According to Grigorovich et al. (13), transdisciplinary research involves collaboration between academic and non-academic sectors and enhances knowledge integration, scientific productivity and capacity, and public involvement in research. In the case that rehabilitation professionals, SUs, and other stakeholders are involved in the execution and implementation of research results, transdisciplinary research could be talked about. When collaboration concerns only professionals, multiprofessional rehabilitation is an exactly descriptive term, and when collaboration concerns only researchers, multidisciplinary research is the exact term.

In Finland, a nationwide strategic research and development program for rehabilitation is lacking (1). In the Action Plan for Reforming Rehabilitation Services 2020–2022 (1), it is stated that the implementation plan and activities related to the development of rehabilitation education, and research appear to be mainly regional. This implies that the capacity of networks and collaboration among researchers and professionals is not fully exploited. In addition, it creates geographical inequality between SUs, as some regions may have better possibilities to develop their rehabilitation practices in line with research activities.

As previously described, nationwide multi-professional rehabilitation requires extensive collaboration in the field. This study bridges rehabilitation practice and research by studying the current and future knowledge needs of professionals and SUs in two different regions in Finland: Jyväskylä and Helsinki. Jyväskylä is located in central Finland, surrounded by sparsely populated rural communes and towns, and Helsinki is located in southern Finland, where the Helsinki metropolitan area consists of a concentration of major cities.

The purpose of the study is to describe the current and future knowledge needs of rehabilitation from professionals' and SUs' perspectives to enhance the implementation of research. This study describes and compares regional knowledge needs in rehabilitation research. The specific study question is: What transdisciplinary rehabilitation knowledge needs do professionals and SUs in rehabilitation practice have?

## Method

### Study Design and Participants

The study is part of the Platform Ecosystem for Strengthening of Research, Development, and Innovation (RDI) Activities in Multidisciplinary Rehabilitation' (REcoRDI) project. REcoRDI aims to improve research result utilization and the impact of applied research on rehabilitation by strengthening network collaboration and developing a roadmap for applied research on multidisciplinary rehabilitation. The project is being conducted in collaboration with JAMK University of Applied Sciences, Jyväskylä, and Metropolia University of Applied Sciences, Helsinki, between April 1, 2019 and March 31, 2022 (14).

The knowledge needs of rehabilitation practice were collected through focus group (FG) discussions, using a qualitative research approach to gain an understanding. FG interviews were selected as a data collection method enabling the construction of information from professionals' and SUs' discussions. An FG is a group of people with interest, expertise, and experience of the research phenomenon (15). According to constructivism as a philosophical approach of science, the views of reality are created through human interactions with the context, and acquired knowledge is socially constructed (16–18). The interest in knowledge was practical (19).

The participants for the FG were recruited through purposive selection from the regional partners of the universities of JAMK and Metropolia. The regional rehabilitation partners of JAMK and Metropolia, were contacted through email and were asked for their consent to take part in the study. The purpose was to gather a group who were (1) multi-professional, (2) multi-sectoral, (3) experienced in multi-professional rehabilitation (at least 1 year), (4) professionals working mainly with rehabilitees, and (5) Finnish speaking. In the Metropolia sample, SUs and their relatives were also included in order to collect the data from their perspectives. The participants are described in Table 1.

**Table 1:** Participants in the group interviews.

**Participants (*n* = 37)**	**Work sector**
Children's neurologist (*n* = 1)	Public
Physiotherapist (*n* = 15)	Public, Private, 3rd sector
Occupational therapist (*n* = 5)	Public, 3rd sector
Speech therapist (*n* = 1)	3rd sector
Psychologist (*n* = 1)	3rd sector
Disability ombudsman (*n* = 1)	Public
Service manager (*n* = 4)	Public
Instructor (*n* = 1)	Public
Development or project manager (*n* = 3)	Public
Teacher (*n* = 1)	3rd sector
Service user (*n* = 4)	Expert by experience

The study was conducted in accordance with the Declaration of Helsinki and good scientific practice. It included no invasive or potentially physically or psychologically harmful elements that would exceed the limits of normal daily life. Due to the COVID-19 pandemic, the interviews were performed online, all participants were informed about the study by email, and they gave their written consent for participation by responding by email. In addition, they received oral information supported by written slides at a Zoom meeting before the interview started. According to Finnish legislation and guidelines of the Finnish National Board on Research Integrity (TENK), an ethical review was not required. A confirmation statement from the TENK is included as Supplementary Material.

### Data Collection and Analysis

The study data consists of nine FG interviews, five conducted by Metropolia in the Helsinki area and four by JAMK in the Jyväskylä area, all organized remotely by the Zoom application between April 2, 2020 and May 5, 2020. Each interview was conducted by two interviewers who were experienced in interviewing and work in JAMK or Metropolia. The first two interviews conducted by Metropolia served as pilots. They were included as research data because they did not justify any significant changes to the themes or the protocol.

There were three to five (typically four) participants in each FG discussion. Five discussions were conducted in the Helsinki area (22 participants) and four in the Jyväskylä area (15 participants), with a total of 37 participants. The audio-recorded interviews lasted 56–89 min (mean time of 69 min). The recordings were transcribed verbatim in a total of 87 pages (Verdana, font size 8, line spacing single), and the transcription service was purchased. The interviews had three main themes concerning (1) current knowledge needs in the practice of rehabilitation, (2) future knowledge needs in the practice of rehabilitation, and (3) utilization of scientific knowledge in the practice of rehabilitation. The themes included discussion about what kind of and what information is needed and why, where information is currently available, and how it is accessed. The participants were asked to discuss future knowledge needs and how that knowledge would ideally be accessed and utilized.

The analysis answered the following questions: (1) “What are the current knowledge needs in applied rehabilitation research?”, (2) “What will be the knowledge needs in applied research in rehabilitation in the future?”, and (3) “What are the needs for utilization of research findings?”. The data produced in Jyväskylä and Helsinki were analyzed separately to identify possible regional differences and similarities.

**Figure 1:**
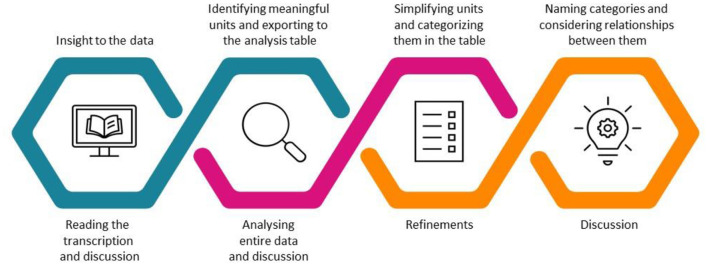
The process of inductive content analysis.

A multi-phased inductive content analysis process (20) was used to analyse the data (Figure 1). First, researchers (3 from Metropolia and 3 from JAMK) read through the transcripts to gain insight into the overall data. The researchers discussed and jointly created a scheme for conducting the analysis. The researchers searched and picked up the meaningful units (phrases) from the transcripts that answered the analysis questions. The units wer e characterized with an identifier and exported as an open code to an analysis table. The open codes were simplified without losing their original meaning and combined with other open codes that included similar content to form sub-topics. In the final phase, similar sub-topics were combined into topics. These identified topics serve a s the basis for the results. For reliability reasons, analysis was carried out separately in JAMK and Metropolia by two research groups that discussed with each other during the process. Second, a separate analysis was performed to detect possible regional similarities and differences. The results were compared as the process progressed and summarized where applicable. The reporting is carried out in two separate studies, referred to as studies 1 and 2, so that important considerations are not missed during the consolidation phase.

## Results

The purpose of the study is to describe the current and future knowledge needs of rehabilitation from professionals' and SUs' perspectives to enhance the implementation of research findings. Due to regional differences, reporting of results is performed separately for the data collected from both areas.

### Study 1, Helsinki Region

#### Current Knowledge Needs

Study 1 brought together transdisciplinary groups of professionals and SUs. Multi-professional practitioners had extensive experience of rehabilitation in different organizations. Their current applied research knowledge needs in multidisciplinary rehabilitation covered six interrelated topics. The topics in which information from research was needed were enabling the meaningful life of SUs; promotion of transdisciplinary collaboration, participation, and inclusion; improvement of development and decision-making; maintaining and demonstrating professional competence to various parties; applying information from research into the practice; and conducting applied research.

Participants highlighted the most diverse factors regarding “enabling an SU as a person to have a meaningful life”. They emphasized the need for a better understanding of a person's life and life situation, as well as for a broad consideration of a person's individual and environmental factors. One participant explained:

“I think that knowledge should improve rehabilitees” life and therefore… Oh, it's difficult to put my thoughts into words… Therefore, there's a need for knowledge about what good life is all about in different situations. The information should be from the rehabilitees' point of view'.

Participants stressed that enabling and strengthening a person's agency is an important issue in rehabilitation, so they need more information on these issues. Enabling rehabilitee's agency is closely linked to interaction and collaboration between professionals, SUs, and other people; therefore, more information is needed about collaboration and interaction in rehabilitation. Digitalisation is now spreading, and more remote rehabilitation is needed. Participants felt that, in order to take advantage of the technology, they should know more about it.

Rehabilitation is always a process, and some processes are more individual and efficient than others. Participants felt that they needed more information on how to create effective individual rehabilitation processes and how to monitor them in day-to-day practices. In addition, they wanted concrete disease-specific rehabilitation guidelines and rehabilitation-related theories to support practical work.

According to the results, the social and health care sector is constantly striving to operate across professional and sectoral boundaries with SUs. Participants felt that they needed more knowledge about the ways, meaning, and impact of transdisciplinary work and how they can strengthen and enable SUs' inclusion and participation.

Participants described that they need scientific knowledge to maintain and demonstrate their professional competence, for example, to other professionals, SUs, and funders of services. Professionals need evidence of the effectiveness and reliability of the methods, for example, to justify their methodological choices or to justify the use of their approach. “Then again, I notice that I always wish for knowledge about effectiveness so that I'm able to work more efficiently with the client”, one participant explained.

The participants also considered the challenges and facilitators of the practical implementation of the knowledge from research. To support the implementation of scientific knowledge, application models, advice and tips created based on tests, and practical examples are needed. It was identified that information is needed on what factors influence the uptake of the data studied, such as the identification and rejection of outdated data. Participants brought up facilitators of the implementation of the research results, such as easy and open access to information, different ways of sharing information, networking, building common understanding and knowledge, and collective utilization of knowledge.

In addition, participants considered how to conduct research on rehabilitation to best meet practical information needs, and stated that at least a variety of methods, approaches, and designs are needed. They considered it very important that professionals, SUs, and relatives are involved in the various stages from research design to reporting and publication, as they know what research information is needed and they are key people in applying information in practice. Technology referred to included using artificial intelligence (AI), algorithms, and bots in searching and getting scientific information that is personalized. In research, there is a need to get data automatically from the everyday life of rehabilitees.

#### Future Knowledge Needs

The future knowledge needs of applied research on interdisciplinary rehabilitation were related to six topics: knowledge on the changing needs of rehabilitation, the individual needs of SUs, rehabilitation as an interactive process between an SU and his or her environment, rehabilitation practices, the effectiveness and benefits of rehabilitation, and supporting and promoting the development and management of rehabilitation.

In the future, knowledge is needed on the changing need for rehabilitation because the problems of SUs have become more complex. The next generations will have more and varying problems than previous generations. Knowledge concerning challenges affecting health and functioning is needed in relation to changing society and the future.

Knowledge that originates from an SU's individual need is needed and meaningful in the future. Individual knowledge arises from the SU's personal needs alongside the diagnosis-specific knowledge. One participant described knowledge that is needed as “Personalized knowledge, in a way, individually for everyone's needs”. In the future, SUs will also need knowledge to promote their ability to function on their own.

It is important to strengthen the knowledge of rehabilitation as an interactive process of the SU and environment. Knowledge about rehabilitation or habilitation processes is needed as well as research about building up and supporting one's agency. In the future, more knowledge is needed about the impacts of the environment—attitudes and physical environment—on the rehabilitation process. As one participant said:

I'd be interested in knowing how people's attitudes and the changing of attitudes affect the rehabilitation process. We'll all encounter some phases in our lives—and I'm wondering how attitudes and atmosphere have an impact on how we all are capable of and able to participate equally in society.

Also, information is needed about enablers of rehabilitation.

There are many knowledge needs concerning rehabilitation practices. In the future, it will be important to increase knowledge about prevention, early-stage guidance, rehabilitation in everyday life, the use of International Classification of Functioning (ICF), peer support, and expert-by-experience services. Regarding the research on remote rehabilitation, digitalisation and technology are highlighted. In the future, knowledge about rehabilitation will be needed for political decision-making on the one hand and for information guidance on the other. The knowledge also needs to include collaboration between professionals as well as interaction between professionals and SUs.

Knowledge about the effectiveness and benefits of rehabilitation is needed in the future. It is important to be able to demonstrate the impacts of rehabilitation as well as provide evidence of the introduction of new technology. It is also important to be able to justify rehabilitation on a personal and societal level.

Knowledge is needed to develop rehabilitation and its administration. Research on strategic management and networks is needed. Research on professionals' knowledge and skills in rehabilitation is important for transferring tacit knowledge. To this end, it is increasingly important that rehabilitation professionals and SUs are involved in the conduction of the study, as described in one discussion: “And I am convinced that information is needed on how SUs are involved in the development of rehabilitation”. Future research should be able to utilize big data from people's daily lives and to study the uptake of research findings. Participants also discussed the use of technology to aid in the implementation of research results.

#### Summary of Study 1

The current and future knowledge needs in Helsinki (Table 2) reflected variability and multiplicity of research subjects, especially concerning better understanding of SUs' individual lives and needs for rehabilitation and active roles and partnerships in rehabilitation. Now and in the future, more knowledge is needed on participation and collaborative frameworks and settings both for research implementations and for rehabilitation practices that are seen as interactive processes. The attitude toward transdisciplinary rehabilitation is obvious, but participants required more understanding about collaborative thinking, activities, and reciprocal communication.

**Table 2:** Summary of study 1.

**Study 1**	
**Current knowledge needs**	**Future knowledge needs**
Enabling meaningful life for service users	Service users' individual needs
Promotion of transdisciplinary collaboration, participation, and inclusion	Rehabilitation as an interactive process of the service user and environment
Improvement of development and decision-making	Changing needs concerning rehabilitation
Maintaining and demonstrating professional competence to various parties	Rehabilitation practices
Applying scientific knowledge in practice	Effectiveness and benefits of rehabilitation
Conducting applied research	Supporting and promoting development and management of rehabilitation

*The current and future knowledge needs in Helsinki*.

Professionals and SUs in the field of rehabilitation need more knowledge about how the information is designed in an individually understandable way to meet the needs of the rehabilitee. In the future, rapid changes in society and technology will put pressure, as well as opportunities for the development of rehabilitation practices. Therefore, more knowledge to support development and decision-making is needed. Knowledge about the effectiveness of rehabilitation and its factors is required in the future. Currently, evidence is used and needed to maintain and demonstrate professional competence to different parties. Technical solutions, digitalisation, and AI were seen as key factors in remote rehabilitation, knowledge implementation, and research. In addition, more transdisciplinary knowledge is needed about practitioners' and SUs' roles in rehabilitation and its research.

### Study 2, Jyväskylä Region

#### Current Knowledge Needs

Study 2 brought together a multidisciplinary group of professionals working in different areas and sectors of rehabilitation, including those working with unemployed people and families. The current knowledge needs of applied research in multidisciplinary rehabilitation covered five interrelated topics. Evidence about methods and best practice is needed in the practice of rehabilitation for several purposes. Knowledge needs concerning other professions is a prerequisite for collaboration within rehabilitation in the health and social service field; relating to the SUs, it is necessary to understand the individual's situation; and regarding societal phenomena, it is needed to understand socially influencing factors. Besides knowledge needs, the utilization of scientific knowledge was also brought up.

Regarding evidence about the effectiveness of methods and best practice, professionals face competing demands within their organization and need to have justification and reasoning internally for their work or work methods, or in situations that require more human or financial resources. For example, one participant explained the need for scientific knowledge as a justification to their employer by saying, ‘All situations where you need to justify your actions, are those. For example, at the workplace, I need to have good support for everything I suggest to the managers. I can't just say “I feel this would be nice”'. Therefore, the requirement for evidence-based practice is essential. Furthermore, some suggested that there may be competing views among colleagues on the “right” methods of rehabilitation, and sometimes reaffirming the overall practices of everyday life is needed. Vocational rehabilitation requires knowledge about SUs' re-employment pathways. Mentioned in particular were vocational re-education, employment-promoting institutions, the labor market and working life, the future of work, changes in occupations, and work ability.

Furthermore, some participants stated that they need knowledge to justify their actions to managers and policy makers. Evidence-based information on effective rehabilitation is also used to influence local welfare policy and the division of resources; this requires evidence about the services. In Finland, rehabilitation services are increasingly provided by the private sector. Among those working in the private sector, the need for knowledge was for marketing purposes, to attract possible SUs, and to have research-based working methods to stand out among other service providers.

The participants brought up evidence of rehabilitation as an important knowledge need in general. This concerned various topics on rehabilitation, methods and measures used to evaluate effectiveness, and best practice. As raised in the interviews, efficient rehabilitation practice includes information on the right timing of rehabilitation. This should be included in the knowledge on rehabilitation services in general. For example, the discussion brought up the justification for organizing preventive work that is often cut down to save financial resources.

As rehabilitation requires collaboration between various professionals, multi-professional work requires knowledge on the collaboration, methods, services, and theoretical foundations of other professional groups, and an extensive understanding of those. Participants reflected on the need to learn and get extensive knowledge on the practices of medicine, psychology, and social work, for example. A physiotherapist may have knowledge needs related to the holistic view of an SU. For example, back pain and fear of pain is the cause-and-effect relationship between body and mind, which needs to be paid attention to. This kind of knowledge builds shared understanding to support work in the overall social and health care services and to serve the needs of the SU.

Various knowledge needs relating to the SUs were considered: individual characteristics are related to the physical condition and health of the SUs, the significance of rehabilitation, and knowledge of the overall life span and various contexts of daily life. Professionals require knowledge to evaluate these as part of their daily work.

Participants brought up the knowledge needs on societal phenomena, such as inequality, socio-economic class differences, knowledge of disadvantaged groups, and unemployment. In their daily work, inequality was noted in the practices of obtaining appropriate services for the SUs. Professionals need extensive knowledge on these phenomena to best support SUs.

The interviews also discussed the utilization of scientific knowledge. Factors that advanced the use of research relate to accessibility of research findings, both in terms of usability and accessing the journals and books where the research is presented in detail. Many of the participants working in small non-research–focused organizations brought up that the lack of sufficient access to knowledge is the greatest obstacle to following research in their professional field. Organizations do not necessarily have resources to pay for the fees to access research publications. Another important factor is that concepts, expressions, and terminology used in research need to be accessible to both professionals and SUs. Participants also raised a concern that scientific knowledge of rehabilitation is fragmented and scattered across various communication channels, leading to difficulties in finding the right information and a delay in the implementation of the scientific knowledge into practice.

#### Future Knowledge Needs

The future knowledge needs covered six interrelated topics. The vivid discussions concerned digitalisation and remote rehabilitation, multi-professionalism, the contexts of rehabilitation, the change process of the individual SU, impact and effectiveness, and work ability and functional capacity. The pace of rapid change in society, such as digitalisation, was also brought up in the discussions, as was urbanization and immigration.

Opportunities offered by digitalisation and technological development, as well as remote rehabilitation, formed essential knowledge needs for the future of rehabilitation research. One participant reflected on the topic by saying:

We are going toward remote rehabilitation. Rehabilitation does not only take place face-to-face with the rehabilitee. Now it would be a good time to study the effectiveness of remote rehabilitation. We need knowledge about what remote rehabilitation is, what it brings, what is good, and its effectiveness.

Some participants called for controlled trials to evaluate the differences in the effects of face-to-face and remote rehabilitation methods. While remote rehabilitation was extensively discussed, digitalisation did not only relate to remote rehabilitation; participants also discussed other developments, such as the use of AI. The participants brought up the need to understand how AI will affect work processes and working with SUs. For example, it was seen as a tool to manage service paths in the complex service system, but the participants called for more information.

The need to understand work and pre-assumptions of various disciplines and across rehabilitation practice in the future was important for the participants. One participant reflected on this saying:

In practice you note, it is great to note this, that “wow! He or she is thinking like that”. And often we have these stereotypes of different professions and what they do. It is really rewarding to note that someone thinks totally different from you. It would be beneficial to know about these presumptions much more.

Rehabilitation takes place in a specific context, and the discussions reflected the need to gain knowledge on the various contexts and significance of rehabilitation. More knowledge is needed on reablement. Participants discussed, for example, what needs to be considered to gain effective results within the reablement process. Significant knowledge needs noted not only concern of the micro-level factors, but also how meso- and macro-level development affects rehabilitation practices. The living environment, such as urbanization and its significance, was raised, as well as inequality more generally.

The core of effective rehabilitation is providing the right services at the right time. This requires from the SU readiness and motivation for the rehabilitation process. At the micro-level, the participants brought up that they need more information on the change process of the individual rehabilitee to gain the best possible outcomes.

#### Summary of Study 2

Current and future knowledge needs in Jyväskylä (Table 3) reflected variability between individuals and society; some participants pointed out knowledge needs relating to individual characteristics while others identified extensive societal themes. Collaboration between various professionals and multi-professionalism, as well as evidence and effectiveness of the rehabilitation services and methods, were seen as issues that require further knowledge currently and in the future. Digitalisation and the context of rehabilitation were mentioned as essential and significant future knowledge needs, but were also mentioned in relation to current needs. Identified challenges about the use of scientific knowledge are related to open access, terminology of reporting, and communication channels.

**Table 3:** Summary of study 2.

**Study 2**	
**Current knowledge needs**	**Future knowledge needs**
Understanding a service user's individual situation	The change process of the individual service user
Societal phenomena	Work ability and functional capacity
Collaboration between various professionals	Multi-professionalism
Evidence about effectiveness of methods and best practice	Impact and effectiveness
Utilization of scientific knowledge	Digitalisation and remote rehabilitation
	The contexts of rehabilitation

*The current and future knowledge needs in Jyväskylä region*.

## Discussion

This study uncovered the current and future knowledge needs of rehabilitation research from the perspectives of professionals and SUs. Two separate studies from two regions, Helsinki and Jyväskylä, showed that there are more common knowledge needs than regional differences. The current and future information needs of both regions are strongly related to enabling and understanding the needs of SU individuals for a meaningful and inclusive life and thus correspond well to current perceptions of good rehabilitation practices (1, 2).

Another general need for knowledge in both areas concerns the promotion of multiprofessional and multidisciplinary rehabilitation and rehabilitation research, as described by Grigorovich et al. (13). The present study made it clear that both professionals and SUs are aware and willing to participate in transdisciplinary collaboration. More knowledge about the implementation of scientific knowledge, evidence and best practice, remote rehabilitation and digitalisation for the improvement of rehabilitation development, research, decision-making, and management are required now and in the future. Knowledge on the involvement of SUs in collaboration on both rehabilitation and research and the conduction of the applied research was more emphasized in the Helsinki metropolitan area than in the Jyväskylä area.

The future knowledge needs in both regions were related to the changing needs of rehabilitation and remote rehabilitation based on rapid change in society and digitalisation. Both results emphasized that more knowledge is needed about the individuality of the SUs and the significance of their environment for rehabilitation. More scientific knowledge is needed on the different rehabilitation practices and contexts that are expected to play an even greater role in the success of rehabilitation in the future. In Jyväskylä, future knowledge needs are also focused on the work and functional capacity of the SUs. In the Helsinki metropolitan area, more information was needed regarding the development and administration of rehabilitation in the future (Table 2).

**Figure 2:**
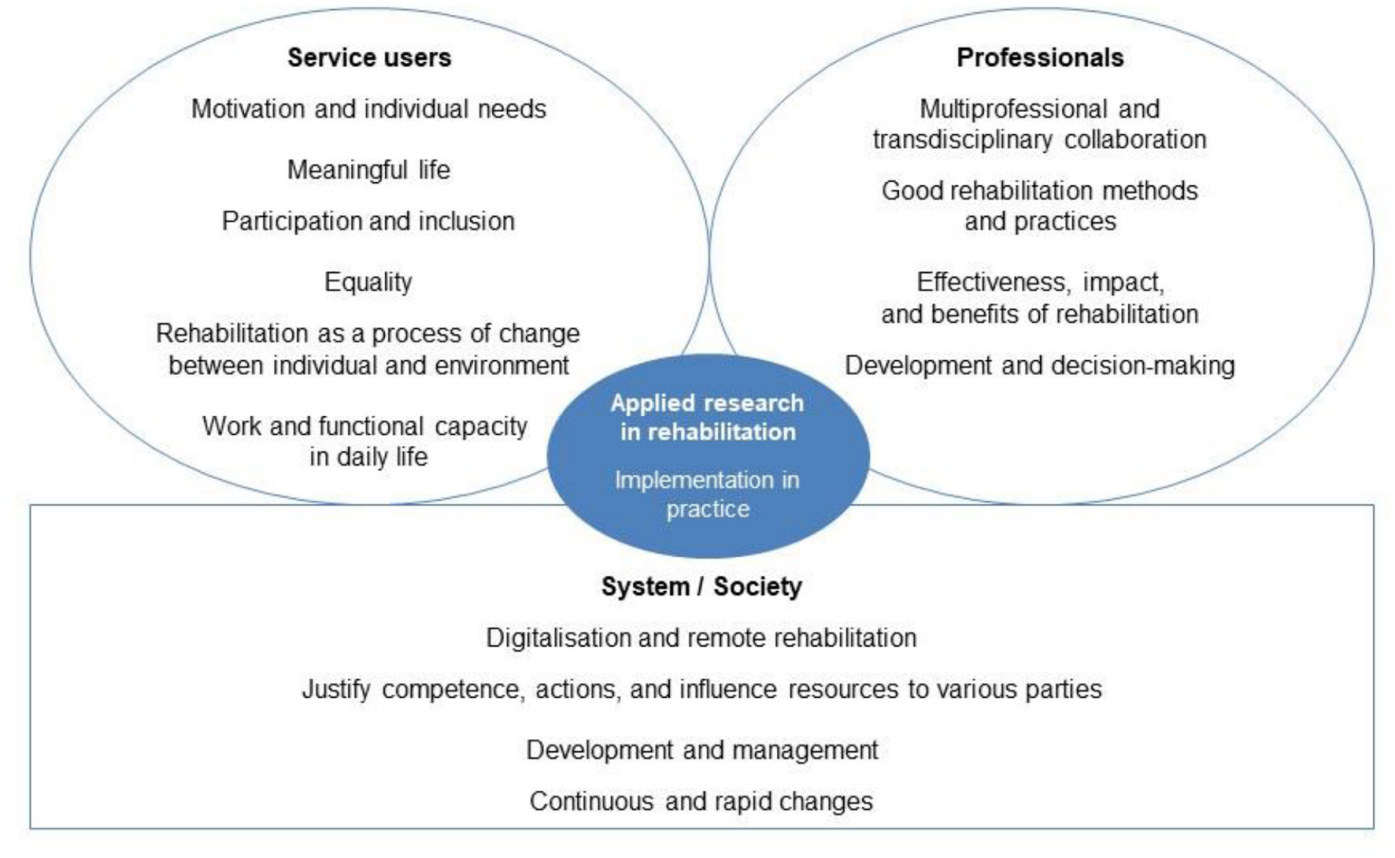
Current and future knowledge needs in rehabilitation research.

Studies 1 and 2 produced similar and complementary results on the current and future information needs of rehabilitation research. In Figure 2, the information needs are grouped into one overall picture and structured into three information user groups. Figure 2 clearly shows how different user groups need research information for different purposes.

Both now and in the future, the scientific knowledge needs in Finnish rehabilitation will be emphasized in justifying the effectiveness, impact, benefits, competence, and use of resources for financiers and other parties. From the perspective of rehabilitation, this is an external need for information that does not directly aim to promote rehabilitation, but to justify existing rehabilitation. The situation is understandable through the ongoing reform of the social and health sector, the shrinking economy in the sector, and the consequent intensification of competition.

The knowledge needs reiterate the importance of an SU's individuality and meaningful life for rehabilitation, as well as the importance of different environments, contexts, and rapid change. Applied rehabilitation research faces major challenges in meeting these information needs. In practice, this requires that professionals and users of practical rehabilitation work be interested in and have the opportunity to participate in the conduction of the study. Therefore, in the future, there will be a need to develop participatory research methods and inclusive research approaches.

Ideally, the knowledge needs of the professionals and SUs should guide the research activities in rehabilitation. The current and future knowledge needs reflected a great variability and multiplicity of research subjects, and what the research needs should thematically address. The use of concepts by the participants may at some instances be less detailed. Some specifications, such as robotisation as part of digitalisation or specific measures at the workplace to enhance employability, may have not been explicitly pointed out.

Scientific knowledge needs of rehabilitation should be viewed at different levels, and at a specific temporal and physical place and setting. First, professionals and SUs contemplated knowledge needs on the individual level. This study confirms the previously known notion that there is a need for research concerning a better understanding of individual needs and environments. For practical purposes, this will require research-based development of sensitive tools and methods for understanding individual preferences and needs. Second, at the professional level, there is a need for effective methods and practices, as well as a need to understand the role of SUs and their ownership of and possibilities to participate in the rehabilitation process. The involvement of the SUs also concerns knowledge creation: there is a need for research knowledge on participatory and democratic methods of knowledge creation, and especially about collaborative knowledge creation and activities, as well as reciprocal communication. Third, there is a knowledge need related to the wider societal level, such as the service environment, and particularly on the marketisation of the service and the austerity politics of public services. Rehabilitation practitioners need to have selling points to promote their work within and outside their organization.

Technical solutions, digitalisation, and AI were seen as key factors both in remote rehabilitation practice and in research. Remote rehabilitation was brought up as an important field of urgent research; not enough is known on the practices and effectiveness of remote solutions, and rapid development of digital tools and programs calls for research on the topic. The interviews took place in the time of COVID-19 that perhaps raised the question of remote services more strongly compared to if there had not been a pandemic.

### Methodological Solutions

Study 1 and study 2 brought together multi- and transdisciplinary groups of professionals, SUs, and students working in different areas and sectors of rehabilitation, including those working with unemployed people and families. They had long experiences of rehabilitation in different organizations. Some of them pointed out knowledge needs relating to individual characteristics, while others further identified extensive societal themes as knowledge needs of professionals. Private sector workers are faced with the demand to respond to the market, while those working in the public sector face municipal austerity politics that they wish to influence. According to Charmaz (21), the criteria for qualitative research data are the richness and adequacy of the data. Regardless of the method, the material should be sufficient and should include views of the subject from various standpoints. The different perspectives of the participants have enriched the research results and helped to look at the research subject in a variety of ways. The data were saturated because no new categories emerged during the last interview's data analysis.

This study has some limitations. Given the multiplicity of professionals working in rehabilitation, and even the ambiguousness of the field, it was not possible to include all professions, which may hinder external validity. A more extensive and versatile selection of informants might have brought up new topics. Some professions (for example physiotherapists) are highlighted in the sample which may emphasize their knowledge needs in the results. However, the focus groups were multidisciplinary including several professions to even out the differences of knowledge needs of certain professions. Another obvious limitation concerns the choice of participants: study 2 included only professionals, while study 1 also included SUs. This is a significant difference between the groups, and its significance in terms of the diversity of perspectives enriches the material. Thus, its impact on the quality of research has been positive rather than restrictive. It should also be noted that in studies 1 and 2, different researchers acted as data collectors, analysts, and reporters. Because researchers' own perceptions can never be completely ruled out, they can be reflected in the content, interpretations, and perceptions of the interviews. To minimize this weakness, there were several investigators in both studies who discussed their interpretations together and, in addition, the investigator groups discussed both interpretations together. As noted, regardless of the differences between the FGs and regions in which the group discussions took place, there were no significant differences between the regional data collections.

Finally, in this study, researchers have extensive experience in practical rehabilitation work. They have surrendered, through critical review and discussion, to an ongoing and long-term process in which previous experiences with perceptions of rehabilitation and its research have provided a background for understanding the phenomena. Scholars have been open to new ideas, which has helped to gradually break the boundaries of previous perceptions and see their shortcomings (22).

Rehabilitation has received increasing global attention (23). It is anticipated that the need for rehabilitation will increase overall in the future. This study has noted the needs of rehabilitation services, education, and research in Finland also, but significant knowledge gaps remain. Future research is needed on the evolving knowledge needs of various actors, including professionals and SUs, and how to best find and meet these needs. In this study, we only touched on the various methodologies of implementing research, and this is an area that should be discussed in future studies. Overall, research on rehabilitation needs to be responsive to the ongoing changes of society.

## Conclusion

According to Peirce (24), the principle of pragmatism is that the value of knowledge is based on its usability and meaning for practice. In light of this maxim, research should provide information particularly for the needs of establishing high-quality rehabilitation and practical development. As a conclusion from the results, rehabilitation research data should serve not only funders, decision-makers, and politicians, but more so practical developers, planners, producers, and users of rehabilitation services. Our research showed that it is particularly important that research results reach professionals and SUs since, besides practical implementation, they play a key role in communicating information to decision-makers and funders. For this reason, it is important that the knowledge needs and involvement of professionals and SUs in the various stages of research preparation and implementation should be strengthened and made possible by researchers and research communities. Thus, strengthening the practical perspective may also have an impact on guiding future research funding.

## Data Availability Statement

The raw data supporting the conclusions of this article will be made available by the authors, without undue reservation.

## Author Contributions

MT, EJ, NV, and TH conducted the interviews. MT, EJ, TH, SH, and SS analyzed the data. All authors contributed to the interpretation of data for the article, contributed to the drafting of the article, revising it critically for important intellectual content, provided final approval of the version to be published, made substantial contributions to the conception or design of the work, and agreed to be named on the author list and approved the full author list.

## Funding

This study was funded by the Ministry of Education and Culture (Grant Number 411287).

## Conflict of Interest

The authors declare that the research was conducted in the absence of any commercial or financial relationships that could be construed as a potential conflict of interest.

## Publisher's Note

All claims expressed in this article are solely those of the authors and do not necessarily represent those of their affiliated organizations, or those of the publisher, the editors and the reviewers. Any product that may be evaluated in this article, or claim that may be made by its manufacturer, is not guaranteed or endorsed by the publisher.

## References

[1] Ministry of Social Affairs and Health. Rehabilitation Reform: Action Plan for Reforming Rehabilitation Services 2020–2022. (Publications of the Ministry of Social Affairs and Health 2020:39) (2020). Available online at: http://urn.fi/URN:ISBN:978-952-00-8443-1

[2] Paltamaa J Karhula M Suomela-Markkanen T Autti-Rämö I Editors. Basis of a good rehabilitation practice. From analysis of current practice evidence to recommendations. A rehabilitation development project for severely disabled persons. Helsinki: Kela (2011). Available online at: http://hdl.handle.net/10138/24581

[3] Morris JH Bernhardsson S Bird M-L Connell L Lynch E Jarvis K. Implementation in rehabilitation: a roadmap for practitioners and researchers. Disabil Rehabil. (2019) 42:22. 10.1080/09638288.2019.1587013 30978129

[4] Sarkies MN Bowles K-A Skinner EH Haas R Lane H Haines TP. The effectiveness of research implementation strategies for promoting evidence-informed policy and management decisions in healthcare: a systematic review. Implement Sci. (2017) 12:392–429. 10.1186/s13012-017-0662-0 29137659 PMC5686806

[5] Banner D Bains M Carroll SL Kandola D Rolfe DE Wong C. Patient and Public Engagement in Integrated Knowledge Translation Research: Are we there yet? Research Involvement and Engagement. (2019) 5:8. 10.1186/s40900-019-0139-1 30805202 PMC6373045

[6] Greenhalgh T Jackson C Shaw S Janamian T. Achieving research impact through co-creation in community-based health services: literature review and case study. Milbank Q. (2016) 94:2. 10.1111/1468-0009.12197 27265562 PMC4911728

[7] Vingerhoets C Hay-Smith J Graham F. Intersection of the elements of evidence-based practice in interdisciplinary stroke rehabilitation: a qualitative study. NZ J Physiother. (2020) 48:31. 10.15619/NZJP/48.3.06

[8] Sackett DL Rosenberg WMC Gray MJA Haynes BR Richardson SW. Evidence based medicine: what it is and what it isn't. BMJ. (1996) 312:371. 10.1136/bmj.312.7023.71 8555924 PMC2349778

[9] Haapakoski K. Good work in Rehabilitation Examination: Abductive research on the incongruity of the ‘good suppositions' in the rehabilitation work (PhD dissertation). University of Jyväskylä (2015). Available online at: http://urn.fi/URN:ISBN:978-951-39-6314-9

[10] Hinkka K Härkäpää K Järvikoski A. Monimenetelmäinen kuntoutuksen tutkimus. Artikkelikatsaus kuntoutuksen 3. valtakunnallisesta tutkimusseminaarista. Online working paper 19/2010. (Helsinki: Kela) (2010). Available online at: http://hdl.handle.net/10138/24203

[11] Korkeamäki J. Kuntoutuspalveluita aikuisten oppimisvaikeuksiin. Opi oppimaan-kehittämishankkeen arviointia. In: Hinkka K, Härkäpää K, Järvikoski A, editors. Monimenetelmäinen kuntoutuksen tutkimus. Artikkelikatsaus kuntoutuksen 3. valtakunnallisesta tutkimusseminaarista. Helsinki: Kela (2010). Available online at: http://hdl.handle.net/10138/24203

[12] Shaw WS Findley PA Feuerstein M. Twenty years of multidisciplinary research and practice: then and now. J Occup Rehabil. (2011) 21:13–21. 10.1007/s10926-011-9339-8 22065200

[13] Grigorovich A Fang ML Sixsmith J Kontos P. Defining and evaluating transdisciplinary research: implications for aging and technology. Disabil Rehabil: Assist Technol. (2019) 14:6. 10.1080/17483107.2018.1496361 30318930

[14] Rantakokko M Sipari S Paltamaa J Malinen K Korniloff K Harra T. REcoRDI. Monialaisen kuntoutuksen soveltavan tutkimustoiminnan vahvistaminen ekosysteemissä. Kuntoutus (2019). Available online at: https://journal.fi/kuntoutus/article/view/97251

[15] Wilkinson S. Focus group research. In: Silverman D, editor. Qualitative research: theory, method, and practice. Thousand Oaks, CA: Sage (2004). p. 177–99.

[16] Guba EG Lincoln YS. Competing paradigms in qualitative research. In: Denzin NK, Lincoln YS, editors. Handbook of Qualitative Research. Thousand Oaks, CA: Sage (1994). p. 105–17.

[17] Kukla A. Social Constructivism and the Philosophy of Science. London: Routledge (2002).

[18] Morgan DL. Focus groups as qualitative research. Newbury Park, CA: Sage (1997). 10.4135/9781412984287

[19] Habermas J. Knowledge and interest. Inquiry. (1966) 9:71–2. 10.1080/00201746608601463

[20] Kyngäs H. Inductive Content Analysis. In: Kyngäs H, Mikkonen K, Kääriäinen M, editors. The Application of Content Analysis in Nursing Science Research. Cham: Springer (2020). 10.1007/978-3-030-30199-6_2

[21] Charmaz K. Constructing Grounded Theory. A Practical Guide through Qualitative Analysis. London: SAGE Publications (2006).

[22] Charmaz K. Grounded theory methods in social justice research. In: Denzin NK, Lincoln YS, editors. Strategies of Qualitative Inquiry. Thousand Oaks, CA: SAGE Publications (2013). p. 291–336.

[23] World Health Organization. Rehabilitation 2030: A Call for Action (2017). Available online at: https://www.who.int/disabilities/care/Rehab2030MeetingReport2.pdf?ua=1 (accessed June 8, 2021).

[24] Peirce CS. The Maxim of Pragmatism (Lecture 1). In The Essential Peirce, Volume 2: Selected Philosophical Writings (1893–1913), ed. Peirce Edition Project. Bloomington: Indiana University Press (1998). p. 133–44.

