# Supplementary material for: Knowledge Needs in Rehabilitation: Perspectives of Service Users and Professionals
Source: Front Rehabil Sci. 2022 May 13;3:858081. doi: 10.3389/fresc.2022.858081 (PMC9397881; doi:10.3389/fresc.2022.858081)
Supplement: Supplementary file 1 [file Data_Sheet_1.PDF]

JAMK University of Applied Sciences ETHICS COMMITTEE

Merja Rantakokko

# To whom it may concern,

Merja Rantakokko at JAMK University of Applied Sciences has asked JAMK ethics committee to provide a description of the ethical review system for research in Finland.

In Finland, research with human participants must comply with the guidelines of the Finnish National Board on Research Integrity TENK[1] *The ethical principles of research with human participants and ethical review in the human sciences in Finland. Finnish National Board on Research Integrity TENK guidelines 2019* (pdf).

JAMK University of Applied Sciences has undertaken to comply with TENK's guidelines. The guidelines do not cover medical research as defined by law (Medical Research Act 488/1999) or other research designs where ethical review is a separate obligation laid down by law.

According to the guidelines, research is to be conducted in such a way that the dignity and autonomy of human research participants is respected and the research does not cause significant risks, damage or harm to research participants, communities or other subjects of research.

Ethical review is to be carried out prior to gathering data, if the research contains one or more of the following factors:

1. Participation in the research deviates from the principle of informed consent. Participation is not, for example, voluntary, or the subject is not given sufficient or correct information about the research.
2. The research involves intervening in the physical integrity of research participants.
3. The focus of the research is on minors under the age of fifteen, without separate consent from a parent or carer, or without informing a parent or carer in a way that would enable them to prevent the child's participation in the research.
4. Research that exposes participants to exceptionally strong stimuli.
5. Research that involves a risk of causing mental harm that exceeds the limits of normal daily life to the research participants or their family members or others closest to them.
6. Conducting the research could involve a threat to the safety of participants or researchers or their family members or others closest to them.

If none of the above factors is met, ethical review is not required.

In Finland, neither legislation nor TENK's guidelines require ethical review by an ethics committee for research based purely on public and published data, registry and documentary data or archive data.

Date and signatures

Chair, JAMK ETHICS COMMITTEE
Leena Liimatainen

Secretary, JAMK ETHICS COMMITTEE
Hanna Kontula

---

[1] The Finnish National Board on Research Integrity TENK is an expert body appointed by the Ministry of Education and Culture and tasked with promoting research integrity and preventing research misconduct in Finland. Further information about the ethical review system in Finland is available at www.tenk.fi.

Tämä dokumentti on allekirjoitettu sähköisesti JAMK Sign-järjestelmällä / This document has been electronically signed using JAMK Sign
Recordi_ Eettisen ennakkoarvioinnin kuvaus_20210615 / 8fd496b0-e9cd-4848-a98c-85c78b8abc24 / sivu/page 1(2)

Tämä dokumentti on allekirjoitettu sähköisesti JAMK Sign-järjestelmällä
This document has been electronically signed using JAMK Sign

Päiväys / Date: 16.06.2021 10:03:33 (UTC +0300)

Jyväskylän Ammattikorkeakoulu Oy
Leena Liimatainen
Kehitysjohtaja, Development Director
Organisaation varmentama (JAMK käyttäjätunnus)
Certified by organization (JAMK user account)
Certified by organization

Päiväys / Date: 16.06.2021 10:45:41 (UTC +0300)

Jyväskylän Ammattikorkeakoulu Oy
Hanna Kontula
Johdon assistentti, Executive assistant
Organisaation varmentama (JAMK käyttäjätunnus)
Certified by organization (JAMK user account)
Certified by organization
